# Influence of operator experience on the complete-arch accuracy and time-based efficiency of three intraoral scanners

**DOI:** 10.1016/j.jds.2024.11.009

**Published:** 2024-11-13

**Authors:** Wei-Chun Lin, Chian-Chuen Lee, Sheng-Yang Lee, Chiao-Yun Peng, Chia-Cheng Lin

**Affiliations:** a School of Dental Technology, College of Oral Medicine, Taipei Medical University, Taipei, Taiwan; b Department of Dentistry, Wan-Fang Hospital, Taipei Medical University, Taipei, Taiwan; c Center for Tooth Bank and Dental Stem Cell Technology, Taipei Medical University, Taipei, Taiwan; d School of Dentistry, College of Oral Medicine, Taipei Medical University, Taipei, Taiwan; e Department of Dentistry, Shin Kong Wu Ho-Su Memorial Hospital, Taipei, Taiwan

**Keywords:** Accuracy, Digital impression, Scan time, Trueness, Intraoral scanner

## Abstract

**Background/purpose:**

The performance of intraoral scanners (IOSs) relies on the operator's skills. However, whether operator experience influences IOS accuracy remains unclear. This study investigated the effect of operator experience on the trueness accuracy and time-based efficiency of IOSs.

**Materials and methods:**

Thirty operators were equally divided into two groups on the basis of their IOS-handling experience. Each operator performed simulation scans of a maxillary model in a training dummy by using three IOSs: CEREC Omnicam, Primescan, and Aoralscan 3. A total of 90 scans were generated, and the scan time for image acquisition and the render time required for an IOS to generate a three-dimensional image were recorded. The trueness of each scan was calculated by comparing with a reference scan obtained from an industrial high-precision scanner. The *t* test and the ANOVA followed by the Tukey post hoc test were used to determine statistical differences. Significance was set at *P* < 0.01.

**Results:**

For the three IOSs, no significant difference was noted in trueness accuracy, scan time, or render time between inexperienced and experienced operators. For both inexperienced and experienced operators, Omnicam had significantly less accuracy and longer scan time than did the other IOSs; the render time was significantly shorter for Aoralscan 3 than for the other IOSs.

**Conclusion:**

Operator experience does not substantially influence the trueness accuracy and time-based efficiency of IOSs; these factors vary across IOS types. The render time for obtaining three-dimensional images is a significant feature for improving the time-based efficiency of IOSs.

## Introduction

Digital impression with an intraoral scanner (IOS) has become increasingly popular in restorative dentistry. The impression procedure is an indispensable step for manufacturing dental prostheses. However, conventional techniques involve drawbacks such as patient discomfort, time-intensiveness, and an inability to amend the impression once the material is set.^1^^,^^2^ Digital impressions can minimize the patient's gag reflex,^3^ and effectively reduce the time and costs for manufacturing the restorations; they also allow for immediate chairside correction of erroneously scanned portions.^4^ Therefore, IOSs have emerged as important tools for dental professionals.

The accuracy and efficiency of IOSs are crucial aspects of clinical performance.^5^, ^6^, ^7^, ^8^ Consequently, numerous researchers have focused on IOS accuracy and time.^9^^,^^10^ IOSs capture dental images and generate digital files by using corresponding software; different technologies are used by different IOSs. For image acquisition, photographic or videographic techniques are used.^11^ For distance-to-object determination, technologies such as active wavefront sampling, active triangulation, and confocal microscopy are used.^12^ For three-dimensional (3D) data reconstruction, the calculating of software algorithm determines the trueness of images generated by an IOS.^13^ The aforementioned differences across IOSs may influence their accuracy and efficiency.

In addition to the hardware and software features of a given IOS device, the operator's execution skill can substantially affect its accuracy and efficiency.^9^ Motel et al. highlighted that the sequence or pattern of scanning can affect the accuracy of intraoral scans.^14^ Oh et al. reported that scanning strategy strongly influences scanning accuracy. Moreover, the orientation and motion of the scanner tip can influence the performance of IOSs.^6^ During the scanning procedure, the operator manages the path, orientation, and speed of the scanner; maintains the distance between the scanner tip and the scanned surface; and decides whether to cut off redundant images or rescan mesh holes: all of these actions can influence the accuracy of scans.^9^

The operator's experience in using IOSs for digital impressions is also believed to be influential to its accuracy. Resende et al. reported that more experienced operators obtain more accurate complete-arch scans.^15^ In their in vitro study on the accuracy of complete-arch implant scans, Gimenez-Gonzalez et al. concluded that experienced operators markedly outperformed inexperienced ones.^16^ Similar results were reported by other researchers.^17^^,^^18^ On the other hand, Canullo et al. have concluded that operator experience exerted no significant effect on the accuracy of IOSs.^19^ Arcuri et al. also reported that operator did not demonstrate significant effect on the IOS trueness.^20^ These findings were corroborated by those of other studies.^21^^,^^22^

As evident from the aforementioned background, whether operator experience influences IOS accuracy remains unclear. Therefore, this study investigated the effects of operator experience on the trueness accuracy and time-based efficiency of three IOSs. The following null hypotheses were made in this study: the operator's experience would exert no significant effect on the (1) accuracy or (2) the scan time of IOSs.

## Materials and methods

**Figure 1:**
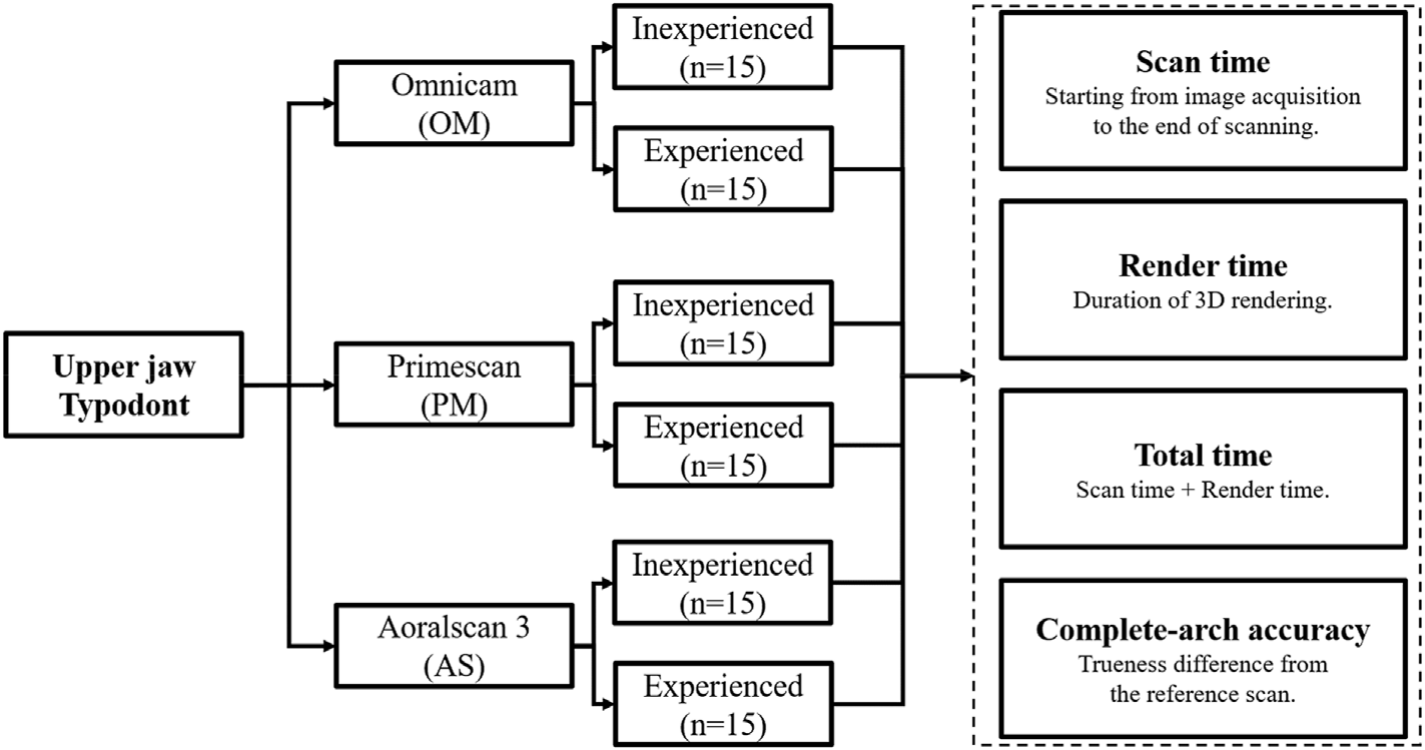
Flowchart depicting experimental workflow.

The study protocol was approved by the Ethics Committee of Shin Kong Wu Ho-Su Memorial Hospital, Taipei, Taiwan (permit number: 20220809R). The experimental workflow is presented in Fig. 1. Thirty operators volunteered for this study. They were equally divided into two groups: the inexperienced group, which comprised dental technology students who had never used any IOS, and the experienced group, which comprised dentists or dental technicians with >2 years of experience in using IOSs regularly. To eliminate potential in vivo confounders, such as tongue movement and saliva on the teeth, we used a standard maxillary dental model (U200, Nissin Dental Products Inc., Kyoto, Japan) for simulation scanning. This model, along with the corresponding mandibular model, was fixed in a dental dummy head. The incisal distance of the two models was set at 45 mm on the basis of the average maximal mouth opening measurements for Asian individuals.^23^

**Figure 2:**
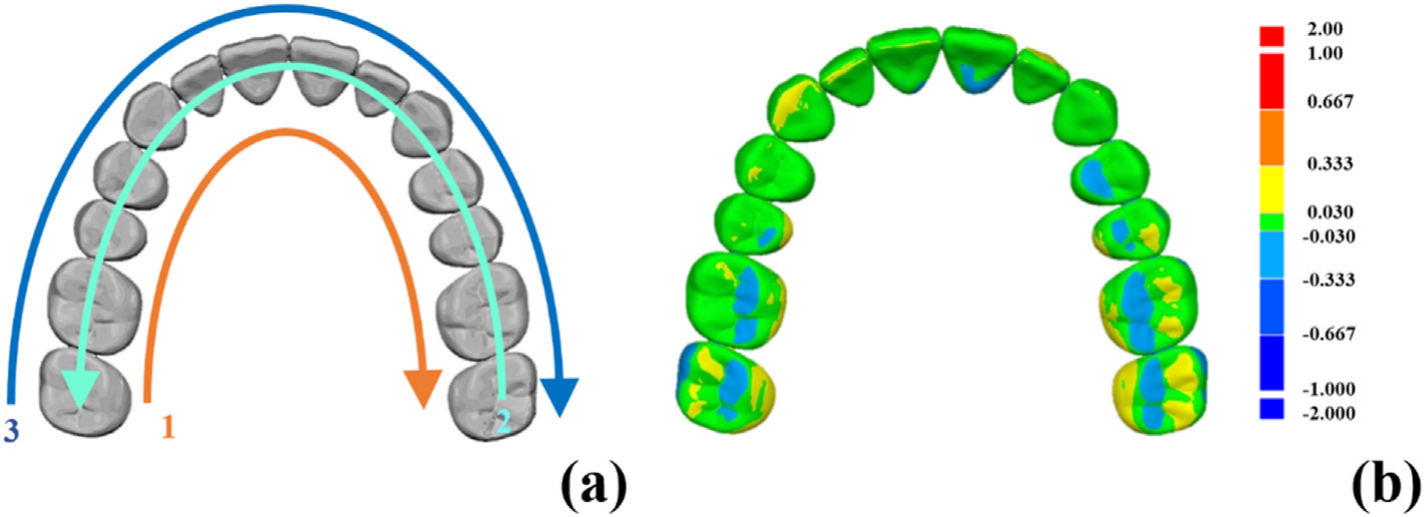
(a) Schematic illustration of the intraoral scanning sequence. Different arrows indicate the scanning order: 1. The orange arrow shows the scan moving from the right to left lingual side. 2. The turquoise arrow indicates movement from the left to right occlusal surface. 3. The blue arrow represents the path from the right to left buccal side. (b) A color map displaying the trueness of a simulated scan compared to the reference scan, where red indicates an increase in deviation and blue indicates a decrease.

The three IOSs used for simulation scanning in this study were CEREC Omnicam (OM; Dentsply Sirona Inc., Charlotte, NC, USA), Primescan (PM; Dentsply Sirona Inc), and Aoralscan 3 (AS; Shining 3D Tech Co., Hangzhou, China). Before the simulation scanning, all participants were provided with relevant instructions on IOS operation. A scanning strategy of the standard maxillary dental model was established, and the participants were instructed to follow this strategy (Fig. 2a). Every operator performed three scans of the typodont with the three IOSs one by one. To reduce potential learning curve effects, three sequences were used for IOS operation: OM→PM→AS, PM→AS→OM, AS→OM→PM, and five participants in each group used a single sequence. The IOSs were calibrated before the experiment and recalibrated after every 15 scans. A total of 90 scans were generated and saved as Standard Tessellation Language files. For the measurement of complete-arch accuracy, an industrial high-precision scanner (ATOS 5; Zeiss GOM GmbH, Braunschweig, Germany) was used; a standard digital file was created as the master model. This model, along with the 90 scan files, was imported into the Medit Compare software (Medit Corp., Seoul, South Korea). The soft tissue portion was trimmed using the smart single tooth selection option; each scan was overlapped with the master file by using the automatic alignment option. The trueness accuracy of each scan was calculated in the deviation display mode (Fig. 2b).^24^

To evaluate the efficiency of each scan, the interval from the start to completion of image acquisition was recorded and considered to be the scan time. Subsequently, the time required for an IOS to generate a 3D image was recorded and considered to be the render time. The total time spent on each scan was the sum of the scan and render times.

Data from a pilot study suggested a sample size of 15 scans per group was required to achieve an effect size of 0.5, power of 80 %, and α-error value of 5 %. Statistical analyses were performed using SAS (version 9.4; SAS Institute, Cary, NC, USA). Quantitative data are presented in terms of mean and standard deviation values. Between-group comparisons were performed using the student *t* test and the one-way analysis of variance test, followed by the Tukey post hoc test to determine significant differences. Significance was set at *P* < 0.01.

## Results

**Figure 3:**
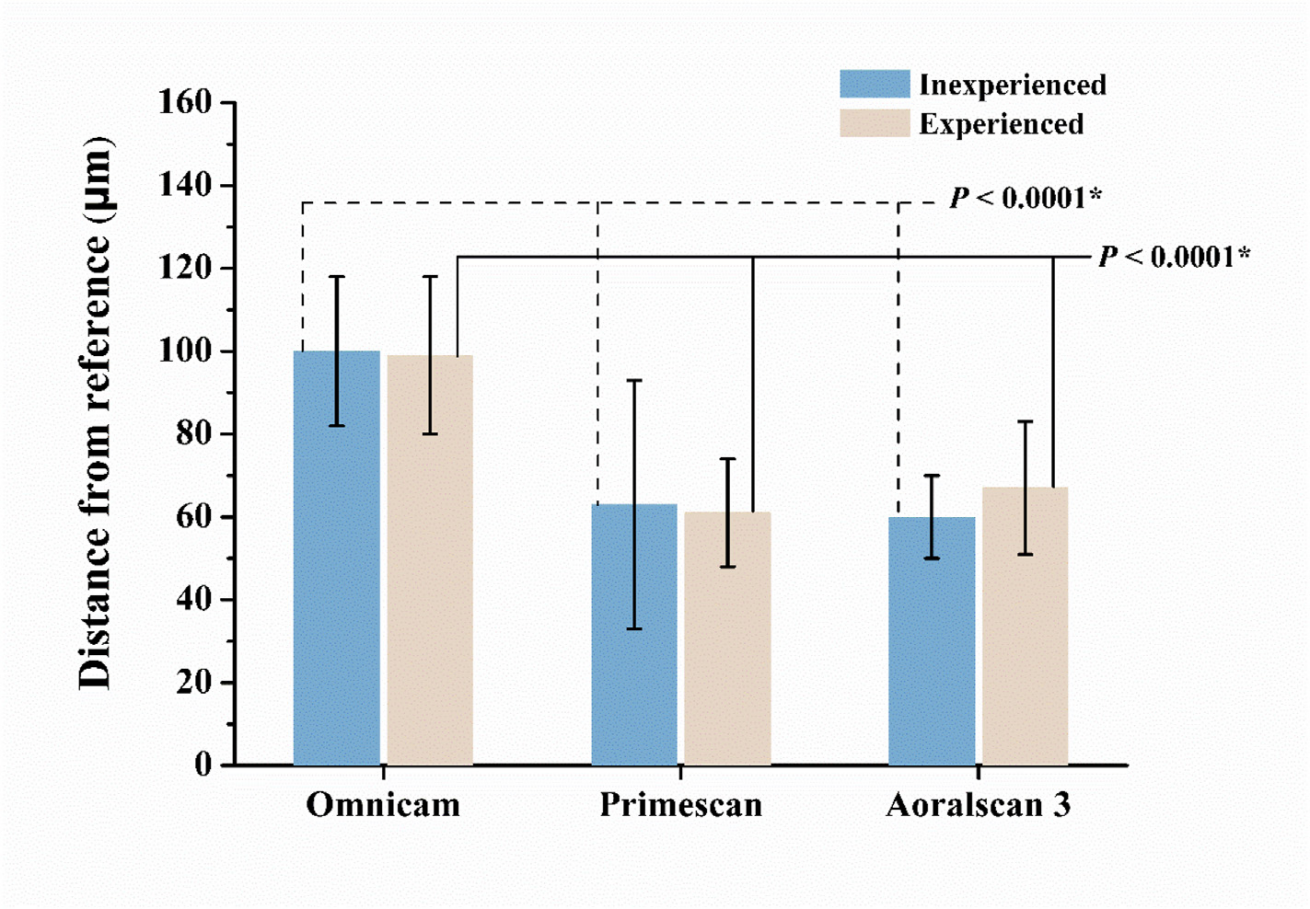
Accuracy of complete-arch scans performed by inexperienced and experienced operators using different intraoral scanners (IOSs). No significant difference was observed between inexperienced and experienced operators for any IOS. However, for both groups, significant differences in trueness were found among IOSs, with Omnicam demonstrating lower accuracy compared to Primescan and Aoralscan 3 (as indicated by the asterisk).

For inexperienced and experienced operators, the trueness accuracy values of OM, PM, and AS were 100 ± 18 and 99 ± 19 μm, 63 ± 30 and 61 ± 13 μm, 60 ± 10 and 67 ± 16 μm, respectively (Fig. 3). No significant difference was noted between inexperienced and experienced operators for any of the three IOSs. However, significant differences were observed among the three IOSs. For both inexperienced and experienced operators, complete-arch accuracy was significantly lower for OM than for PM and AS (*P* < 0.0001).

**Figure 4:**
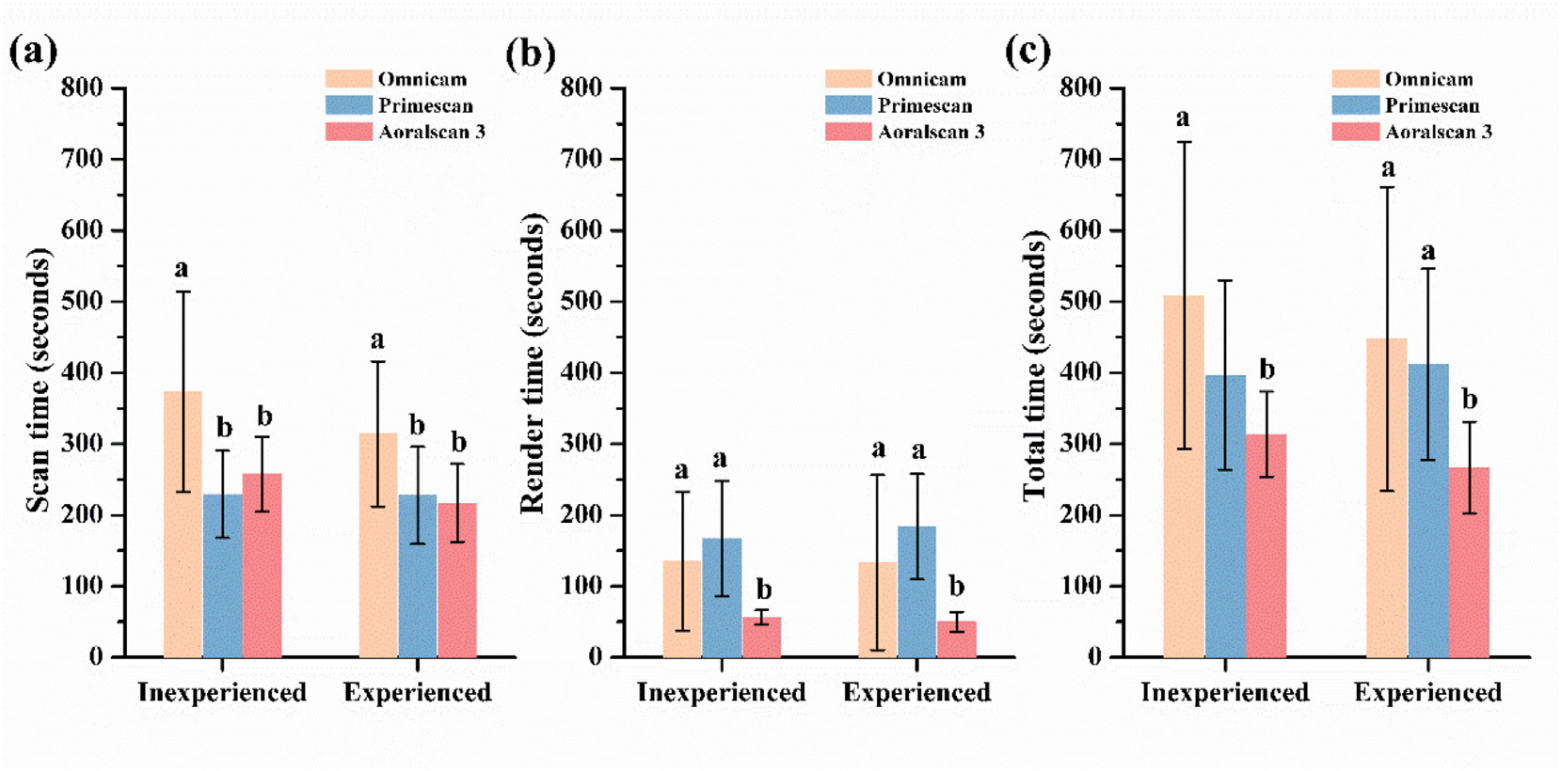
Scan time (a), render time (b), and total time (c) for complete-arch scans performed by inexperienced and experienced operators using different intraoral scanners (IOSs). No significant difference was observed between operator groups. Significant differences among IOSs are indicated by different lowercase letters (a, b; *P* < 0.01).

The scan time and render time for inexperienced and experienced operators using different IOSs are presented in Table 1. No significant difference was observed between inexperienced and experienced operators in scan time and render time, regardless of the IOS used. However, significant differences were noted among the IOSs for both inexperienced and experienced operators. Scan time was significantly longer for OM than for PM and AS for both inexperienced (*P* = 0.0003) and experienced (*P* = 0.0023) operators. Render time was significantly longer for OM and PM than for AS for both inexperienced (*P* = 0.0005) and experienced (*P* = 0.0003) operators. The total time was significantly longer for OM than for AS for inexperienced operators (*P* = 0.0038) and for OM and PM than for AS for experienced operators (*P* = 0.0049) (Fig. 4).

**Table 1:** Scan times (seconds) of inexperienced and experienced operators for different intraoral scanners.

Group		Omnicam	Primescan	Aoralscan 3	*P* value
Inexperienced	Scan time	373.4 ± 140.7^a^	229.4 ± 61.3^b^	257.5 ± 52.9^b^	0.0003∗
	Render time	135.4 ± 97.7^a^	167.3 ± 80.9^a^	56.1 ± 10.2^b^	0.0005∗
	Total time	508.8 ± 215.9^a^	396.7 ± 132.7	313.7 ± 59.6^b^	0.0038∗
Experienced	Scan time	314.1 ± 102.0^a^	227.8 ± 68.2^b^	217.0 ± 54.8^b^	0.0023∗
	Render time	133.4 ± 123.0^a^	184.1 ± 74.3^a^	50.0 ± 14.3^b^	0.0003∗
	Total time	447.5 ± 213.8^a^	411.9 ± 134.3^a^	267.0 ± 64.6^b^	0.0049∗

∗ Indicates significant difference (*P* < 0.01).

Lowercase letters indicate significant differences between different scanners.

## Discussion

Accurate IOS-based digital impressions are crucial for dental treatment. The accuracy of an IOS is associated with its hardware and software systems. Because the operator determines the pattern and procedure of scanning, the performance of an IOS and thus the accuracy of the scan is thought to be influenced by operator experience. However, our findings suggest that operator experience does not significantly influence the accuracy of IOSs. Thus, our first null hypothesis was accepted.

The literature presents inconsistent data on the effect of operator experience on the accuracy of IOSs. Resende et al. analyzed these effects by enrolling only three operators with different levels of experience.^15^ Gimenez-Gonzalez et al. assessed the effects by enrolling only two operators with >4 years of experience in using IOSs and two without any experience.^16^ Overall, regardless of their results, studies exploring the effect of operator experience have had a small number of operators.^17^^,^^18^ When the number of enrollers is small, operators’ personal skills, rather than their experience, are likely to influence the results. According to Gimenez et al., the accuracy of IOS is influenced by the operator; however, this effect is not necessarily related to operator experience.^25^ To alleviate the effect of personal skills, we recruited 30 participants to perform simulation scans. This approach reduced the influence of interpersonal differences; thus, our results closely reflect the real-world situation and are convincing.

Zarauz et al. evaluated the effect of age on the accuracy of scans performed by inexperienced operators.^26^ The researchers found that older operators may require extended training to develop increased accuracy and that newer IOS software can increase the accuracy of scans performed by inexperienced operators. We recruited young students as inexperienced operators. These students were somewhat familiar with information technology products, which made IOS operation easy for them. Revell et al. investigated the correlation between complete-arch implant scanning and operator experience and found that operator experience enhanced the trueness of the edentulous mucosa but not the implant deviation.^18^ This result suggests that if the scanning procedure is more difficult, operator experience plays a more significant role. In the present study, the participants scanned the dentition of a standard model, which was an easy procedure relative to scanning prepared abutment teeth or implant scan bodies. This may explain why the inexperienced operators achieved satisfactory accuracy.

For the three IOSs, no significant difference in scan time and render time was noted between experienced and inexperienced operators. Thus, the second null hypothesis was also accepted. However, the findings revealed that trueness accuracy and time-based efficiency were dependent on the type of IOS. Similar results were reported by Kim et al., who found that scan time was associated with IOS type but not operator experience.^27^ Lim et al. indicated that the trueness and precision of newer IOSs were better and less likely to be influenced by clinical conditions.^28^ Furthermore, Zarauz et al. highlighted that newer software increased the trueness of scans performed by inexperienced operators.^26^ Revilla-Leon et al. suggested that the influence of operator experience on IOS accuracy was more pronounced when older-generation systems were used.^9^

Two of the three IOSs used in this study (OM and PM) were produced by the same manufacturer. PM, the newer-generation IOS, was faster and more accurate than OM was, which is reasonable. OM had significantly less trueness accuracy and longer scan time than did PM and AS. According to Gomez-Polo et al., scanning trueness decreases with an increase in the number of rescanned mesh holes.^29^ For current IOSs, a longer scan time is inevitably associated with a higher number of rescans. Our findings also implied that longer scan times can be associated with lower trueness accuracy.

Regarding the total time required for a complete scan, AS was significantly faster than OM was for both inexperienced and experienced operators; however, PM was not significantly different from OM. Because PM and AS did not significantly differ in terms of scan time, the faster total time for AS may be attributable to its shorter render time. Nevertheless, the trueness accuracy of AS was consistent with that of PM. Research indicates that software improvements can enhance time-based efficiency.^30^ Software-based technologies have become increasingly important because IOS manufacturers try to reduce hardware size for efficient clinical handling.^13^ Current devices with powerful artificial intelligence technologies can automatically identify and remove soft tissue artefacts. Furthermore, software features such as “smart stitching,” which uses artificial intelligences to stitch together data, have improved IOS efficiency.^31^ Therefore, 3D rendering, or image postprocessing, is a critical factor for improving the time-based efficiency of IOSs. Manufacturers should optimize 3D rendering software, as the render time is crucial for enhancing overall efficiency.

This study has some limitations. First, we could not measure the learning curve effects for individual operators; however, the sequence of IOS use was adjusted to ensure equal learning opportunities for every operator in each type of IOS. Second, the in vitro nature of this study limited our ability to interpret the results. Finally, the level of experience was defined in an all-or-none manner, which may be oversimplification. Further in vivo studies are required to evaluate the influence of varying levels of experience on the accuracy and efficiency of IOSs and to validate the present in vitro results.

Within the limits of this study, we draw the following conclusions. First, operator experience does not significantly influence the trueness accuracy and time-based efficiency of IOSs. Second, the type of IOS influences its accuracy and time-based efficiency. Finally, render time is a crucial feature for improving the time-based efficiency of IOSs.

## Declaration of competing interest

The authors declare no conflicts of interest relevant to this study.
